# An attention based deep learning model of clinical events in the intensive care unit

**DOI:** 10.1371/journal.pone.0211057

**Published:** 2019-02-13

**Authors:** Deepak A. Kaji, John R. Zech, Jun S. Kim, Samuel K. Cho, Neha S. Dangayach, Anthony B. Costa, Eric K. Oermann

**Affiliations:** 1 Icahn School of Medicine at Mount Sinai, Department of Orthopaedics, New York, NY, United States of America; 2 Icahn School of Medicine at Mount Sinai, Department of Neurological Surgery, New York, NY, United States of America; 3 Icahn School of Medicine at Mount Sinai, Department of Neurology, New York, NY, United States of America; Clemson University, UNITED STATES

## Abstract

This study trained long short-term memory (LSTM) recurrent neural networks (RNNs) incorporating an attention mechanism to predict daily sepsis, myocardial infarction (MI), and vancomycin antibiotic administration over two week patient ICU courses in the MIMIC-III dataset. These models achieved next-day predictive AUC of 0.876 for sepsis, 0.823 for MI, and 0.833 for vancomycin administration. Attention maps built from these models highlighted those times when input variables most influenced predictions and could provide a degree of interpretability to clinicians. These models appeared to attend to variables that were proxies for clinician decision-making, demonstrating a challenge of using flexible deep learning approaches trained with EHR data to build clinical decision support. While continued development and refinement is needed, we believe that such models could one day prove useful in reducing information overload for ICU physicians by providing needed clinical decision support for a variety of clinically important tasks.

## Introduction

The Society for Critical Care Medicine (SCCM) estimates that 55,000 patients per day are treated in intensive care units (ICUs) throughout the United States at an annual cost of approximately $82B USD in 2005 [1]. These patients have an average length of stay of 3.8 days with a mortality rate of 10-29% [1, 2]. One of many significant challenges faced by physicians managing these patients is the need to deal with a tremendous amount of real-time information. It is important to prevent information overload to ensure safe and efficient delivery of patient care. Reducing information overload is associated with more rapid care with fewer errors [3]. To aid in clinical care and provide high level supportive analytics, numerous attempts have been made to develop and implement predictive models and computer assisted diagnostic (CAD) solutions that are interpretable, generalizable, and able to predict important clinical outcomes [4–8].

The standard for computer assisted decision support has primarily consisted of scoring systems. These scoring systems are based on some type of feature selection followed by logistic regression and/or hazard regression. It is common for such clinical algorithms to yield suboptimal results because they are usually trained on one outcome (such as risk of death) but are utilized for another (such as being able to predict early clinical deterioration) for which they were not designed [6, 7, 9]. Furthermore, such models may not be be able to learn the non-linear relationships that frequently exist in medicine and biology. Despite the strengths of machine learning models, they are difficult to implement due to their reliance on large datasets which are rare in healthcare. Moreover, these models often exhibit limited interpretability, which have driven the adoption of transparent linear models [10, 11].

Although scoring systems such as APACHE, SOFA, and qSOFA are still most common for evaluating morbidities in the ICU [4, 7, 12], machine learning approaches are becoming increasingly used in the literature. Single layered multilayer perceptron networks have proven useful for predicting survival, length of stay, extubation in the ICU setting [13–15]. However, these networks have required manually curated feature selection *a priori* and do not take advantage of the full selection of features routinely gathered in the ICU setting, thus ignoring the potential for complex non-linearly constructed latent features which may not be individually correlated with the target endpoint. Additionally, none of these methods offer the ability to deal with time-varying inputs. While we identified studies that used Bayesian artificial neural networks and logistic regressions on time-series data in the ICU, both studies featurized the series as individual, uncorrelated feature inputs [16, 17]. By featurizing each time step as independent, the model is not able to contextualize each window in relation to previous ones.

Deep learning based models are revolutionizing how many fields approach problems that involve large amounts of rapidly changing, high-dimensional data [18]. Neural network and recurrent neural network (RNN) models for predicting clinical events have been found to be more accurate than other approaches, but are not easily interpreted [19–23]. However, recently developed soft attention models offer the promise of providing interpretability while retaining the flexibility and versatility of deep learning approaches. Such models were initially applied to predict outpatient disease progression [24, 25]. They have subsequently been applied to predict mortality, length of stay, and diagnoses (particularly sepsis) using EHR data [26–28]. The Attend and Diagnose model of Song et al. used a novel self-attention mechanism to improve an RNN’s predictive accuracy for mortality, decompensation, LOS, and disease state, but did not explore interpretability [29]. The most relevant prior model, RETAIN from Choi et al., predicted outpatient heart failure and used attention at the level of two parallel RNNs to interpret visit- and variable-level importance [24]. While important time points were easily extracted from this model, identifying variables that were important at a given point in time required additional calculation [24].

We hypothesized that it would be possible to predict a variety of clinical endpoints in the ICU by training an RNN to routinely available variables from a critically ill patient’s ICU admission to sequences of those endpoints, and that the incorporation of variable-level attention could promote straightforward interpretability of such a model to clinicians. This approach is analogous to how intensive care physicians iteratively analyze and update patient management in the ICU during daily rounds, basing their management decisions on the most important daily findings, which they identify from the massive amount of available data, in combination with the patient’s clinical trajectory over the previous days. We demonstrate that when combined with a variable-level attention mechanism, RNNs can offer interpretability directly at the level of input variables rather than at the level of an embedded space which is more challenging to interpret. With data from the Medical Information Mart for Intensive Care III (MIMIC-III) critical care database, we use this approach to predict three clinical events: sepsis, myocardial ischemia, and administration of the antibiotic vancomycin.

## Materials and methods

### Ethics statement

Our dataset was constructed by processing the MIMIC-III Clinical Dataset, a critical care database from Beth Israel Deaconess Medical Center (BIDMC). Construction and de-identification of MIMIC-III were approved by the BIDMC and MIT institutional review boards (IRBs) [30]. Their documentation states that the “[r]equirement for individual patient consent was waived as the study did not impact clinical care and all data were de-identified”. As the study utilized a publicly available dataset, there was no need for further local IRB approval for this research. All pre-processing, data analysis, and machine learning were performed in accordance with MIMIC-III guidelines and regulations.

### Data description and cohort construction

The MIMIC-III dataset is comprised of de-identified health data from critical care patients with features such as demographic data, vitals, labs, and medications, often with minute to minute resolution. The full preprocessing and analysis pipeline will be made available on Github (https://github.com/deepak-kaji/mimic-lstm/) to facilitate reproducibility.

Individual patient ICU admissions 2 days or longer were identified (n = 56,841); this number was larger than the number of patients as some patients had multiple stays. ICU stays longer than 14 days were truncated to the first 14 days. When a patient’s stay was fewer than 14 days, extra days were masked out with 0 in training, and these masked days were excluded from test calculations. Padded time steps were later masked out by the model. For each day a patient was in the ICU, three daily target variables were constructed: (1) myocardial infarction (MI), defined as troponin > 0.4 ng/dL in patient without an ICD-9 consistent with CKD (2) sepsis, defined as two SIRS criteria (Heart Rate > 90; Respiratory Rate > 20; WBC (White Blood Cell Count) > 12,000 or WBC < 4,000; Temperature (F) > 100.4 or Temperature (F) < 96.8) in a patient with ICD-9 consistent with infection (3) vancomycin administration, defined as receipt of any dose of vancomycin. These three targets were chosen to highlight three different medical outcomes driven by lab findings, vital signs, and medications, respectively. As these were calculated on a daily basis, a patient could have different target values on different days of their stay depending on each day’s events.

Predictor variables were similarly constructed for each day of each patient’s ICU stay. Features were identified from the MIMIC-III chartevents based on their known clinical relevance to our target endpoints, and variables for which >=75% of values were missing were excluded; missing values were imputed using the variable median. Due to the presence of erroneously recorded values in the MIMIC-III dataset, we censored values above the 95% percentile and replaced these with the median value. In total, 119 features (S1 Table) were available for further analysis. These included complete blood count (CBC) with differential, vital signs, lab results, demographic data, and prescribed medications. These 119 initial features were processed into 225 predictor variables using straightforward statistical transformations of real-valued variables (average, max, min, std) and conversion to indicator variables of categorical variables (e.g., 1 when medication recorded as administered, otherwise 0). Quality control was performed on labs and vitals to remove erroneous values from the MIMIC-III and identify clear outliers as described above. Normalization was performed by subtracting the mean and dividing by the standard deviation for all 225 variables. These scaled variables were used as predictors for the model [31]. All preprocessing was performed in Python 3.6.

Certain predictor variables that solely determined targets (troponin in myocardial infarction modeling, vancomycin administration in vancomycin modeling) were censored from those models to avoid learning trivial solutions. Specifically, the MI model contained 221 features, the sepsis model contained 225 features, and the vancomycin model contained 224 features.

For all experiments, 70% was designated for training, 10% for validation, and 20% for test. Models were trained exclusively on the training set and the validation set was used for hyperparameter tuning only. Results were reported on the held-out test data exclusively. It was necessary to reduce class imbalance in training data to learn informative models. Test data was not artificially balanced in any way. In sepsis and vancomycin modeling, some cases completely negative for the target variable over the full ICU course were removed from training data to achieve equal balance between cases containing and cases lacking at least one positive day over the ICU course. In myocardial infarction modeling, only cases positive for the target in at least day over the ICU course were included; this was necessary to achieve convergence to an informative model, as even in this group most ICU days were negative for the target variable.

### Model background

An LSTM is a type of RNN that incorporates a highly effective mechanism for determining which elements of the encoded state to pass forward to the next cell at each point in time and which to use to predict the target variable [32]. While the basic RNN is a useful network for dealing with sequential information, the LSTM cell has certain distinct advantages over simple RNN units. Simple RNNs tend to exhibit degradation when learning long input sequences where the network does not have an opportunity to reset its internal state. Simple RNNs can also fail to predict outcomes when the most critical information in the sequence is many time steps away from the time window being predicted [33]. However, modern LSTM cells contain input, output, and forget gates. These forget gates allow the network to learn long sequences, to handle long-range dependencies, and to converge on meaningful solutions.

LSTMs have been extended through the incorporation of attention mechanisms, which were initially explored as a method to improve the accuracy of machine translation [34]. The attention mechanism in this paper was adapted from Philippe Rémy’s Github repository [35]. An attention vector learns weights *a*_*i*_ corresponding to features *x*_*i*_ in order to focus the next layer of the model on certain features. In most examples in which attention is used with LSTMs, the *a*_*i*_ are trained to correspond to the embedded state of the LSTM cell at each point in time, and a vector of the cell’s internal state weighted by the learned attention weights is passed forward to the output layer of the network. Attention mechanisms were incorporated into machine translation models primarily to improve performance rather than interpretability, which was a secondary benefit. In the case of medical data, direct interpretation of such an embedded space is challenging. Our features included categorical and continuous variables that were not easily converted into unique discrete inputs, as the vocabulary of a natural language processing application can be. In order to facilitate interpretability at the level of input variables, we learned weights **W**_**k**_ to calculate attention **a**_**k**_ for each of the *k* features **x**_**k**_ (rather than feature embeddings) across time steps:

$$a_{k}=softmax\;(W_{k}x_{k})$$

where each **x**_**k**_ represents a single feature over time (i.e., in the case of 14 daily measurements, a vector of length 14). The time series of input features were weighted by this learned attention vector before being made available to the LSTM as input **y**_**k**_:

$$y_{k}=a_{k}⊙x_{k}$$

### Model development

Our RNN was implemented in Keras with a TensorFlow backend [36]. 3-dimensional data with patient ICU stays (n = 56,841), time steps (n = 14), and features (n<=225) served as input to the attention layer described above. This input layer feeds into a attention layer that weights the inputs. This weighted input layer is fed to a masking layer to contend with masked sequences when patients have ICU stays less than 14 days. Finally, the output of the masking layer is fed directly to our LSTM layer. An LSTM layer with 256 units was connected to a hyperbolic tangent activation function. The output of our network architecture features one dense neuron with a softmax activation to output the probability of a given event over each day of an ICU stay. Activation maps consisting of the raw softmax activations **a**_**k**_ for each input variable k were obtained from the attention layer of our RNN for the purposes of interpreting model outputs.

### Model training

While all of our models employed the same architecture, hyperparameter optimization differed between all three target endpoints. Before manipulating the optimizer’s hyperparameters (learning rate, rho, epsilon, and decay), training set balance, the number of units in an LSTM layer, and feature normalization were all manually tuned first. All hyperparameters were selected prior to model training and were subsequently adjusted in the next train based on the accuracy and AUC of the model on the validation set. RNNs were trained with a RMSProp optimizer with a learning rate of 0.001, rho of 0.9, epsilon of 1e-08 and no decay were used in all models [37]. Batch sizes of 16 were used for all models. MI models were trained for 13 epochs, sepsis models for 17, and vancomycin models for 14 epochs. Binary cross-entropy defined the loss function for all models.

### Evaluation of target modeling

We report AUCs for these three targets for a same-period model that has access to the predictor variables for a given day when predicting targets for that day. We separately report AUCs for these targets for a next-day model that uses the same trained model to make predictions, but masks predictor variables at each time period such that only data from prior days is used to predict each day’s targets. We note that only the latter is a predictive task in which the target occurs after the predictor variables are available.

**Fig 1:**
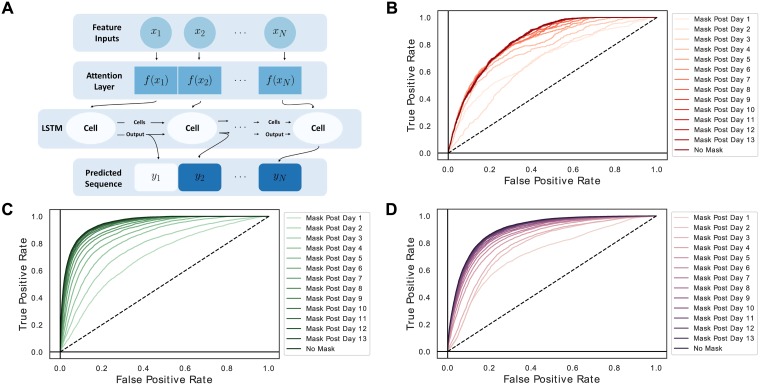
LSTMs incorporating attention can model multiple clinical targets. (A) The schematic of a basic attention-LSTM sequence to sequence architecture. The input variable-level attention layer (highlighted in red) is passed to a recurrent neural network LSTM cell which then sends both an encoded cell state vector and an encoded output to the next time point, the latter of which is used to make a prediction for the target at each time point. (B-D) Three AUROC curves demonstrate this approach’s ability to model same-day myocardial ischemia (AUC 0.834), sepsis (AUC 0.952), and vancomycin administration (AUC 0.904) in the ICU. We note that since predictors and targets are drawn from the same time window in this formulation of the model, this is a modeling rather than predictive task.

We evaluate the ability of our approach to model each same-day target by calculating AUCs with various levels of data availability over time. Reported AUCs were calculated for targets over all available days weighted equally (up to 14). Then the last day’s prediction inputs were hidden while predictions were still made on all possible days. This continued until all but the first day’s predictor inputs were hidden (‘Day 1’), and predictions were still made for all possible days (Fig 1). This allowed visualization of classification performance over the full time series as a progressively reduced length of data was available.

**Fig 2:**
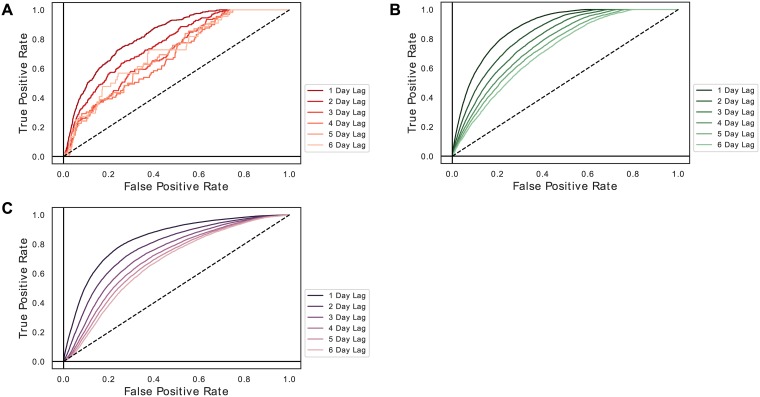
Next-day predictive performance varied depending on the target and location in the time series. (A) Sepsis (next-day AUC 0.876) shows a clear trend towards improved predictive performance later in the course, while (B) vancomycin administration (next-day AUC 0.833) is predicted similarly well over most time periods. (C) Myocardial infarction (next-day AUC 0.823) is rare in later time periods.

A similar process was followed to visualize next-day classification performance over each time step. Only Day 1 predictors were made available, and predictive AUC was calculated for Day 2; then Day 1 and 2 predictors were made available, and predictive AUC was calculated for Day 3; this procedure was followed to make next-day predictions for up to 13 timesteps (Fig 2). The same-day and next-day calculations for PPV and sensitivity were performed similarly, but with a threshold of 0.5.

### Evaluation and visualization of attention

**Fig 3:**
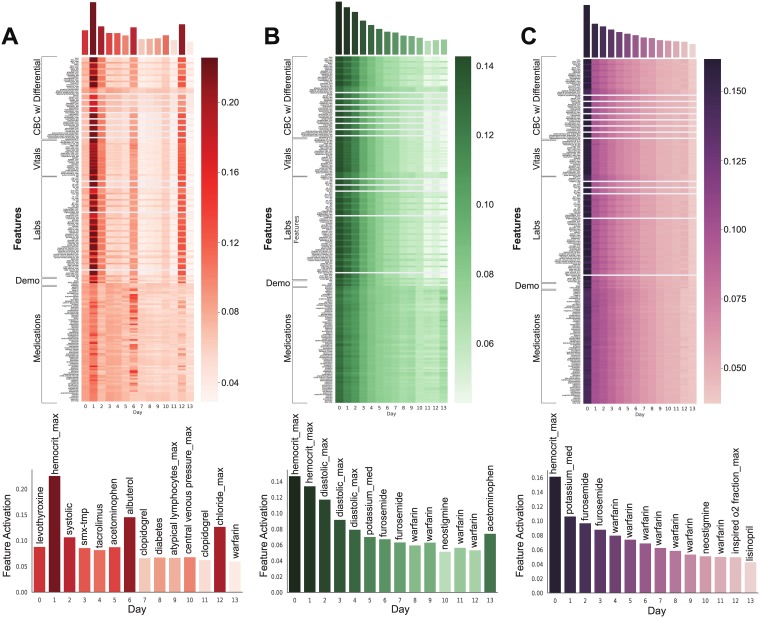
Attention heat maps averaged over all patients with daily attention and features with highest mean activation per day. As described in “Extracting clinical information from average attention” in Methods, heatmaps are second order tensors produced by averaging over all patients. These patient-averaged heatmaps contain activations for each feature at each time step. One-dimensional feature-averaged activations are plotted above each heatmap and reveal days which were most highly attended to by the model. Lastly, each panel contains the activations for the feature which was most heavily attended to at every time step. The patient-averaged attention across all patients with (A) myocardial ischemia, (B) sepsis, and (C) vancomycin, demonstrates emphasis on early days, reflecting the fact that modeling initiation days are critical for making accurate predictions over the time series.

**Fig 4:**
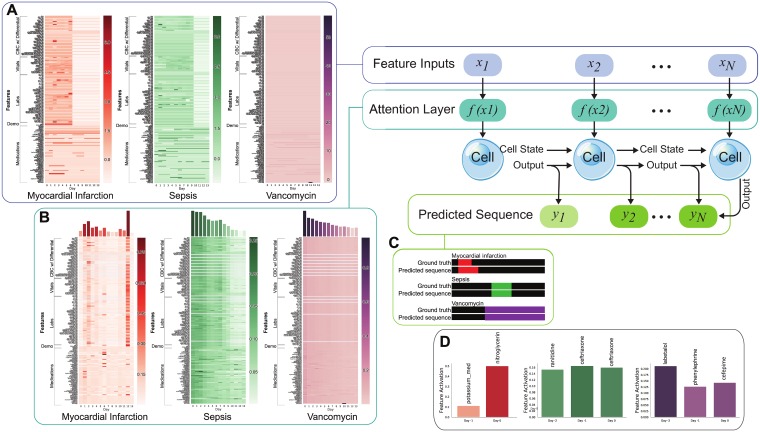
Personalized attention maps for three patients whose ICU courses were positive for myocardial ischemia, sepsis, and vancomycin. Normalized input features in (A) are fed to the attention mechanism whose activations are visualized in (B). Ground truth labels and predictions for all target endpoints are labeled in (C). The days preceding the day of the ICU event, features with the highest time-relative activation on that day were identified in (D). The attention mechanism on the day of MI revealed a focus on nitroglycerin administration. For sepsis, administration of ranitidine and ceftriaxone were highly attended to in the days preceding the ICU event. Finally, in the case of vancomycin, the model attended heavily to labetalol, phenylepherine, and cefepime leading up to vancomycin requirement.

The attention activations over the 14-day time window were used to construct heatmaps for models trained to each of the three targets. These softmax activations summed to one over the full ICU course for each predictor variable, and could be averaged over all test patients to obtain patient-averaged attention maps demonstrating when individual predictor variables had the most influence on each target (Fig 3). Furthermore, these second order patient-averaged attention tensors can be further averaged over the features dimension to obtain the one-dimensional feature-averaged attention vector. This feature-averaged vector (which has also been patient-averaged) reveals the average attention being placed on each day in a 14-day time window. The second order patient-averaged attention map can also be used to extract features with the highest proportional activation in every time step (Fig 4). Fig 4 illustrates similarly constructed activation heatmaps for example patients and demonstrates how such heatmaps can identify factors that might intuitively be expected to predict the target. Fig 4 also identifies the most influential predictor variables for these selected individuals using these attention activations. These were constructed by identifying the features whose activations were highest 48 hours prior to, 24 hours prior to, and the day of the first incident day.

## Results

### Mapping of clinical sequences to events

From a full starting dataset of 56,841 patients, the model was trained to predict sepsis (36,176 patients), myocardial infarction (1,538 patients), and treatment with vancomycin (28,996 patients) using each respective training set. As described previously, training sets were balanced such that the number of patients who experienced an episode of sepsis or a dosage of vancomycin were equal to the number of patients who did not. However, the training set for myocardial infarction contained only patients who experienced an infarct.

In same-period target modeling, the model identified patients with sepsis at an area under the receiver operating characteristic curve (AUROC) of 0.952, patients with myocardial ischemia at an AUROC of 0.834, and treatment with vancomycin at an AUROC of 0.904 (Fig 1B and 1C). The positive predictive values (PPV) for these models were 0.03 for myocardial infarction, 0.74 for sepsis, and 0.69 for vancomycin. The sensitivity was 0.56 for our MI model, 0.73 for sepsis, and 0.71 for vancomycin. For next-day target modeling, the model identified patients with sepsis at an AUROC of 0.876, patients with myocardial ischemia at an AUROC of 0.823, and treatment with vancomycin at an AUROC of 0.833 (Fig 2). The PPV for these next-day models were 0.02 for MI, 0.50 for sepsis, and 0.60 for vancomycin. These models exhibited next-day sensitivities of 0.68 for MI, 0.57 for sepsis, 0.61 for vancomycin. Due to the large size of the MIMIC-III, models were able to converge with fractions of the original training data set (S2 Fig). The same-period and next-day myocardial infarction models had the weakest AUROCs on the test set. The MI model training set required discarding sequences from individuals who did not experience an MI. As described previously, this was required for convergence. However, this negative impacted the PPV of the model at our threshold. Of course, MI may be more challenging to model given our feature inputs and the relatively smaller size of the MI training set.

### Extracting clinical information from average attention

After identifying the existence of these clinical signatures, we sought to understand whether day to day fluctuations in routine ICU variables or baseline admission variables on day 0 were responsible for driving the model’s decisions for each clinical outcome. We examined the activations from the attention layer which helped understand not only the predictive features of the model but also which time steps were predictive for each clinical outcome. If our models were simply learning the distribution of MI, sepsis, and vancomycin treatment by day, we would expect the baseline features from day 0 and their distributions to drive the predictive power. All endpoints have target test distributions that are skewed heavily towards the early time points with MI having the most kurtosis (kurtosis of 1.29 with 66.4% of positive events occurring between day 0 and 2) (Fig 3a–3c and S1 Fig).

### Identifying an individual patient’s relevant clinical factors

Individualized predictions and attention maps can be generated for each ICU patient for each of our targets. We show heatmaps for three patients with their respective ground truth values and predictions for each of our targets: sepsis (Fig 4a), MI (Fig 4b), and vancomycin treatment (Fig 4c). Additionally, we identify the feature which has been the most disproportionately attended to 48 hours before, 24 hours before, and the day of the ICU event.

## Discussion

Improving detection and/or optimizing management of each of the three targets we chose to model—sepsis, MI, and the need for starting medications such as vancomycin—are critical steps towards improving ICU outcomes [3–6, 38]. Sepsis is an important cause of morbidity and mortality for critically ill patients. In-hospital MI increases the risk of mortality and length of stay. Timely initiation of vancomycin can help improve patient outcomes in patients who suffer from MRSA infections.

We found that an LSTM incorporating attention was able to achieve informative AUCs in modeling these targets same-period and predicting next-day. We note that these AUCs cannot be directly compared to the performance of traditional scoring systems as they do not measure only performance in making a new diagnosis on a patient, but rather daily classification performance over a full whole ICU course. We can see in Fig 2 that models generally benefited from access to a longer prior time series. This is intuitive in that more data is available to inform predictions, and is likely somewhat exaggerated due to the fact of missing data. If important data is missing in one time period, but is present in others, this will create additional, if artificial, modeling benefit from longer historical data.

The AUROC, PPV, and sensitivity for the same-day sepsis model suggest that same-day sepsis prediction was the most successful endpoint predicted in our study. Sepsis was defined using SIRS criteria and an ICD-9 code for infection. Since the components used to calculate SIRS criteria were inputs to the model, the model’s relatively strong performance was to be expected. Moreover, the sepsis training dataset contained a myriad of antibiotics to signal infection. The same-day vancomycin requirement model was likely the second strongest using the same criteria. While vancomycin was screened as an input in the vancoymycin requirement model, the presence of other antibiotics in prior days served as excellent proxies. These inputs were highly attended to in the same-day model (Fig 3) and it is likely that they improved next-day vancomycin prediction as well. Models trained towards predicting same-day and next-day MI exhibited lower AUROCs, PPVs and sensitivities. This is likely due to the lack of strong predictors for MI such as EKG findings or chest pains. MI was defined by an elevated serum troponin and therefore MI was difficult to distinguish from demand ischemia which also displays an elevated serum troponin. Models for sepsis and vancomycin were successful, in large part, because medications that were indicative of physician response to infection served as strong model inputs. However, many medications used to treat MI are also part of long term treatment for coronary artery disease, thus obscuring the ability the model to detect a genuine infarction. These factors likely contributed to relatively inferior performance.

Machine learning models could be of great value in providing timely decision support to ICU clinicians, distilling the information overload that is typical of the environment down into the most relevant factors for that patient in any given moment. In this study, we demonstrated how a deep learning model for clinical events in the ICU could be made more interpretable through the incorporation of a variable-level attention mechanism. Interpretability is critical in the healthcare setting. Supervising providers are ultimately responsible for every action that is taken and will demand to understand any recommendation suggested by clinician decision support before implementing it. In additional to their potential for clinical decision support, such models can also suggest promising avenues for future research by identifying potentially non-intuitive and important predictive variables from a wide variety of inputs.

While some maintain that deep learning models are impenetrable ‘black boxes’, we believe that this view is excessively pessimistic [22]. As Lipton has noted, deep learning can facilitate interpretation by “learn[ing] rich representations that can be visualized, verbalized, or used for clustering” [39]. The continued integration of attention mechanisms into clinical deep learning models provide a promising avenue to incorporate a degree of interpretability to clinicians.

While a number of RNN based models to predict clinical outcomes have incorporated attention, we are aware of only two that used attention to identify the variables driving predictions [24, 26]. The model of Rajkomar et al. integrates attention to identify variables but does not describe the specific mechanism used. RETAIN from Choi et al. uses an innovative factorized approach to calculate attention over both variables and time using embedded features rather than the immediate input features themselves; its performance is demonstrated on monthly EHR data to predict heart failure. Neither of these presents data from the ICU setting, which we believe to be a particularly promising use of this real-time variable identification due to the overwhelming amounts of information available and the critical decisions that must be made in real time. We believe that this paper can serve to highlight how an interpretable RNN-based decision support tool could be made for the ICU, as well as demonstrating how attention can be applied at the level of input variables themselves. We note that our variable-level attention approach is atypical in that most attention mechanisms work with embedded features and take an implicit weighted average over such embedded features. Further work is needed to determine if variable-level attention as implemented in this work can be successfully extended to more complicated architectures that simultaneously attend over both time and variable. Either such an approach, or further extension of RETAIN, could offer a path forward for creating interpretable time series models of clinical events.

One advantage of the current approach over traditional scoring systems is its extensibility: we were able to model three substantially different clinical targets utilizing the same framework. One of the common misuses of existing scoring systems is to clinically extrapolate them for targets that they were not trained to predict. For example, APACHE II scores regularly fail to predict mortality [4, 38], and HEART scores do not predict acute MI, but they are generalized to these tasks because clinicians do not have predictive models specifically trained for these tasks. A LSTM-based model incorporating attention could be extended to predict many different variables simultaneously, providing physicians with more tailored and relevant clinical risk prediction tools.

The attention maps in Figs 3 and 4 demonstrate how such maps can identify dynamics of the clinical course. For example, vancomycin is typically prescribed for longer periods of time. Accordingly, we found the model attended most strongly to the first day, since vancomycin prescribed on this day would likely be continued throughout a multiple-day ICU course in a critically ill patient. In contrast, patients rarely maintain elevated troponins for longer than a few days in the ICU, and we found more variation in attention over the course of variables related to MI. Predicting the onset and the termination of signal were critical for modeling these processes. Interestingly, known risk factors for MI such as age or a history of cardiovascular events were not as heavily attended to as features from other subcategories. We attribute this to the fact that all patients included in this group experienced an MI, and in such a positively selected group such risk factors will no longer be predictive. Furthermore, time-varying features are more important for predicting ICU events over such short frames.

The average MI model identified max hematocrit as the most specifically attended-to variable on the most attended-to day, Day 1. This could reflect the fact that troponin leaks (transient elevations in troponin due to stress on the heart, not reflecting a true myocardial infarction) are common in patients who are severely anemic or who are septic and receiving IV fluids that would cause dilutional anemia [40, 41]. This reflects a difficulty in defining variables in sufficiently specific ways to exclude other similar clinical entities (e.g., MI vs demand ischemia). Max hematocrit was also heavily attended to in both the sepsis and vancomycin models, likely reflecting that these patients received significant IV fluids indicated by a sepsis protocol that would similarly lead to dilutional anemia [42]. This suggests that models can attend to interventions made by physicians that relate to the onset of the ICU event.

Our models attended to many features which have known relationships with our target endpoints. In our MI model, the attention mechanism highlighted nitroglycerin (Fig 4), a potent vasodilator, which is part of MI treatment regimes [43, 44]. Nitroglycerin is also indicated for painful angina which is a hallmark of ischemic heart disease. Our model highlighted variance in platelet counts and variance in anion gap the day after the initiation of the MI as significant. Platelet counts are known to fall after the thrombosis of MI [45], and metabolic acidosis can occur after severe MI [46].

The pathological basis for sepsis and vancomycin requirement both begin with an infection. It is likely that ICU patients who required vancomycin were at risk for sepsis or were given vancomycin to help treat sepsis. It is not surprising that the attention mechanisms for both sepsis and vancomycin identified antibiotics such as ceftriaxone and cefepime (Fig 4) as important. In the case of sepsis, the presence of multiple antibiotics that were highly attended to may suggest that intensivists required additional antibiotics or that previous first-line therapies were ineffective in managing infection. The attention paid to ranitidine (an H2 blocker that can protect against gastric ulcers) in our individually highlighted septic patient may reflect that stress ulcer prophylaxis is often given to severely ill ICU patients, including those who are septic. For our patient who required vancomycin, we identified administration of phenylephrine, a pressor used to increase blood pressure, the day prior as particularly significant. This is consistent with a hypotensive septic patient requiring pressor support in whom antibiotics are being broadened to include vancomycin to address an insufficiently treated infection. Vancomycin is frequently added to cephalosporins like cefepime (attended to on Day 1) to cover methacillin-resistant staph aureus (MRSA) [47, 48].

Our attention mechanism not only attended to features which indicated infection, but also to physiological events which were the direct result of infection. For example, daily maximum diastolic pressure was identified at multiple time points for sepsis (Fig 3). While only systolic pressure is a component of qSOFA criteria [49–51], diastolic pressure is required for calculation of mean arterial pressure, a component of SOFA and Apache II scores [12, 52]. Moreover, these scoring systems were used to predict morbidity and mortality from patients already diagnosed with sepsis. Septic shock occurs when sepsis devolves into a vasodilatory shock where pressors are required to maintain mean arterial blood pressure. While diastolic blood pressure has been investigated as a predictor of survival in patients with septic shock, it has not yet been studied as a predictor of sepsis itself. In the context of sepsis, it is possible that decreases in diastolic pressure may signal the onset of septic shock and that increases are signaling the delivery of IV fluids required to maintain mean arterial pressure. Delivery of IV fluids can also explain changes in daily maximum hematocrit (Fig 3). Lisinopril, an ACE inhibitor, and furosemide, a loop diuretic, both decrease blood pressure. Their high activations in our attention maps may reflect that these medications are often used in patients who are hypotensive due to sepsis from infection (Fig 3). The attention paid to warfarin may reflect the fact that anticoagulants would be held in the setting of severe infections that induce coagulopathy [53].

Variable-level attention allowed us to directly identify influential features used by the LSTM in predictions, and the cases we highlight above demonstrate how the models can exploit clinician decision-making in making predictions. In the case of MI, we censored troponins, but the model was still readily able to learn that nitroglycerin suggested a new MI. In the case of sepsis, administration of cephalosporins revealed infection. In vancomycin administration, despite censoring the medication itself, the use of pressors or other antibiotics suggested that vancomycin would be added. While the LSTM appeared to correctly learn the association between these variables and the respective targets, models based primarily on such inputs would not make for useful real-world clinical decision support. A model based on such factors would simply reflect the clinician’s previous assessments back at them. The LSTM’s ready identification of these associations highlights a substantial challenge in creating deep learning-based clinical decision support. Due to the flexibility of deep learning-based approaches, such models may learn to exploit clinician decision-making in making predictions if such decisions are implicit in the data used for training. Considerable thought must be given to mitigating the impact of clinician decisions on model predictions if we are to create useful deep learning-based clinical decision support based on EHR data.

Our study has several limitations. We use one-day timesteps, which is a relatively coarse level of time resolution. A higher level of time resolution is needed for optimal clinical decision support in the ICU, where critical decisions are made throughout the day. This level of time resolution was chosen in order to provide sufficient data for a traditional LSTM to successfully train. Alternative approaches to handling missing data, such as the imputation-free method demonstrated by Lipton et al. on a PICU dataset, may allow successful training at a smaller time interval [54]. A higher level of time resolution would allow for sufficient data to make predictions at clinically important future time intervals.

Despite this attempt to reduce the requirements for data by grouping clinical course into days, a large amount of data was still required due to the flexibility of the relationships LSTM RNNs can learn. The massive MIMIC-III dataset on which the model was trained was difficult to develop and maintain; it would be very difficult to a hospital system to replicate their data curation effort. Even in this high-quality dataset, we note that there was a high degree of missing data. Furthermore, many variables that clinicians might incorporate into their medical decision making were not available for incorporation into a model.

The lack of true out-of-sample testing is a significant limitation of this work. Deep learning models can learn very specific relationships, and have been shown to successfully fit random labels [55]. It is imperative that models are rigorously validated in different patient populations and settings to fully assess their generalizability. While the lack of external validation is common to many such modeling attempts, it is critical that such validation is performed prior to potential deployment [4, 6, 24, 56].

Finally, we note that attention as implemented in this paper does not provide immediately interpretable directional information about a variable. Attention vectors can highlight whether or not a predictor is important, but one cannot identify whether or not a variable increases or decreases the probability of the target event without additional analysis. Recent work on contextual decomposition shows a promising ability to determine the directional effect of input variables in the NLP context and could be extended to this healthcare context [57]. We also recognize that it is difficult for clinicians to interpret high-dimensional attention heatmaps in the hospital. While further research on optimal visualization methods are required, heatmaps may be an effective means of generating hypothesis for future investigation.

In conclusion, we believe that LSTMs with variable-level attention mechanisms trained to ICU data could one day be used to create interpretable decision support for intensivists. We have demonstrated that such an approach can learn informative models for MI, sepsis, and vancomycin administration, and have shown how the individual variables underlying these predictions can be explored using an attention mechanism. However, our results suggest that such models readily learn to exploit variables reflecting clinician decision-making, illustrating a challenge that arises when using flexible deep learning approaches trained on EHR data to build clinical decision support.

## Supporting information

S1 Fig Distribution of total positive days and positive initiation days for total, test, and train datasets. The distribution of total positive days (left) and the distribution of initiation days (right) tend are right skewed for MI (A), sepsis (B), and vancomycin (C) total datasets. However, test data, is a randomly selected 20% of total data, does not perfectly resemble the train data. This suggests the model learned more than just distribution.
(EPS)

S2 Fig Model training with progressive dataset reduction. Decreasing percentages of the training data were used to train models and find convergence. Model AUROCs exhibited only mild decreases with less than 20% of training data for MI (A), sepsis (B), vancomycin (C).
(EPS)

S1 Table Table of MIMIC-III features. These are features from the complete blood count with differential, vitals, lab values, demographics, and medications.
(DOCX)
