# Supplementary material for: An attention based deep learning model of clinical events in the intensive care unit
Source: PLoS One. 2019 Feb 13;14(2):e0211057. doi: 10.1371/journal.pone.0211057 (PMC6373907; doi:10.1371/journal.pone.0211057)
Supplement: S1 Table — These are features from the complete blood count with differential, vitals, lab values, demographics, and medications. (DOCX) [file pone.0211057.s003.docx]

# Supplementary Materials

Supplementary Materials and Methods

Table S1. Final feature list.

| **Supplemental Table 1** | | |  |
| --- | --- | --- | --- |
| Complete Blood Count | | | RBCs |
|  |  |  | WBCs |
|  |  |  | platelets |
|  |  |  | hemoglobin |
|  |  |  | hemocrit |
| Differential | |  | atypical lymphocytes |
|  |  |  | bands |
|  |  |  | basophils |
|  |  |  | eosinophils |
|  |  |  | neutrophils |
|  |  |  | lymphocytes |
|  |  |  | monocytes |
|  |  |  | polymorphonuclear leukocytes |
| Vitals |  |  | temperature (F) |
|  |  |  | heart rate |
|  |  |  | respiratory rate |
|  |  |  | systolic blood pressure |
|  |  |  | diastolic blood pressure |
|  |  |  | pulse oxymetry |
| Labs |  |  | troponin |
|  |  |  | HDL |
|  |  |  | LDL |
|  |  |  | BUN |
|  |  |  | INR |
|  |  |  | PTT |
|  |  |  | triglycerides |
|  |  |  | creatinine |
|  |  |  | glucose |
|  |  |  | sodium |
|  |  |  | potassium |
|  |  |  | chloride |
|  |  |  | bicarbonate |
|  |  |  | blood culture |
|  |  |  | urine culture |
|  |  |  | surface culture |
|  |  |  | sputum culture |
|  |  |  | wound culture |
|  |  |  | inspired O2 fraction |
|  |  |  | central venous pressure |
|  |  |  | PEEP set |
|  |  |  | tidal volume |
|  |  |  | anion gap |
|  |  |  | daily weight |
|  |  |  | tobacco |
|  |  |  | diabetes |
|  |  |  | history of cardiovascular events |
| Demographic | |  | age |
|  |  |  | gender |
|  |  |  | black |
| Medications | |  | epoetin |
|  |  |  | warfarin |
|  |  |  | heparin |
|  |  |  | enoxaparin |
|  |  |  | fondaparinux |
|  |  |  | aspirin |
|  |  |  | ketorolac |
|  |  |  | acetominophen |
|  |  |  | insulin |
|  |  |  | glucagon |
|  |  |  | potassium |
|  |  |  | calcium gluconate |
|  |  |  | fentanyl |
|  |  |  | magnesium sulfate |
|  |  |  | dextrose |
|  |  |  | ranitidine |
|  |  |  | ondansetron |
|  |  |  | pantoprazole |
|  |  |  | metoclopramide |
|  |  |  | lisinopril |
|  |  |  | captopril |
|  |  |  | statin |
|  |  |  | hydralazine |
|  |  |  | diltiazem |
|  |  |  | carvedilol |
|  |  |  | metoprolol |
|  |  |  | labetalol |
|  |  |  | atenolol |
|  |  |  | amiodarone |
|  |  |  | digoxin |
|  |  |  | clopidogrel |
|  |  |  | nitroprusside |
|  |  |  | nitroglycerin |
|  |  |  | vasopressin |
|  |  |  | hydrochlorothiazide |
|  |  |  | furosemide |
|  |  |  | atropine |
|  |  |  | neostigmine |
|  |  |  | levothyroxine |
|  |  |  | oxycodone |
|  |  |  | hydromorphone |
|  |  |  | fentanyl citrate |
|  |  |  | tacrolimus |
|  |  |  | prednisone |
|  |  |  | phenylephrine |
|  |  |  | norepinephrine |
|  |  |  | haloperidol |
|  |  |  | phenytoin |
|  |  |  | trazodone |
|  |  |  | levetiracetam |
|  |  |  | diazepam |
|  |  |  | clonazepam |
|  |  |  | propofol |
|  |  |  | zolpidem |
|  |  |  | midazolam |
|  |  |  | albuterol |
|  |  |  | ipratropium |
|  |  |  | diphenhydramine |
|  |  |  | 0.9% sodium chloride |
|  |  |  | phytonadione |
|  |  |  | metronidazole |
|  |  |  | cefazolin |
|  |  |  | cefepime |
|  |  |  | vancomycin |
|  |  |  | levofloxacin |
|  |  |  | ciprofloxacin |
|  |  |  | fluconazole |
|  |  |  | meropenem |
|  |  |  | ceftriaxone |
|  |  |  | piperacillin |
|  |  |  | ampicillin-sulbactam |
|  |  |  | nafcillin |
|  |  |  | oxacillin |
|  |  |  | amoxicillin |
|  |  |  | penicillin |
|  |  |  | SMX-TMX |
